# Protein kinase C promotes restoration of calcium homeostasis to platelet activating factor-stimulated human neutrophils by inhibition of phospholipase C

**DOI:** 10.1186/1476-9255-6-29

**Published:** 2009-10-30

**Authors:** Gregory R Tintinger, Annette J Theron, Helen C Steel, Riana Cockeran, Lynette Pretorius, Ronald Anderson

**Affiliations:** 1 Department of Internal Medicine, Faculty of Health Sciences, University of Pretoria, South Africa; 2 Medical Research Council Unit for Inflammation and Immunity, Department of Immunology, School of Medicine, Faculty of Health Sciences, University of Pretoria and Tshwane Academic Division of the National Health Laboratory Service, Pretoria, South Africa

## Abstract

**Background:**

The role of protein kinase C (PKC) in regulating the activity of phospholipase C (PLC) in neutrophils activated with the chemoattractant, platelet-activating factor (PAF, 20 and 200 nM), was probed in the current study using the selective PKC inhibitors, GF10903X (0.5 - 1 μM) and staurosporine (400 nM).

**Methods:**

Alterations in cytosolic Ca^2+^, Ca^2+ ^influx, inositol triphosphate (IP_3_), and leukotriene B_4 _production were measured using spectrofluorimetric, radiometric and competitive binding radioreceptor and immunoassay procedures, respectively.

**Results:**

Activation of the cells with PAF was accompanied by an abrupt increase in cytosolic Ca^2+ ^followed by a gradual decline towards basal levels. Pretreatment of neutrophils with the PKC inhibitors significantly increased IP_3 _production with associated enhanced Ca^2+ ^release from storage vesicles, prolongation of the peak cytosolic Ca^2+ ^transients, delayed clearance and exaggerated reuptake of the cation, and markedly increased synthesis of LTB_4_. The alterations in Ca^2+ ^fluxes observed with the PKC inhibitors were significantly attenuated by U73122, a PLC inhibitor, as well as by cyclic AMP-mediated upregulation of the Ca^2+^-resequestering endomembrane ATPase.

Taken together, these observations are compatible with a mechanism whereby PKC negatively modulates the activity of PLC, with consequent suppression of IP_3 _production and down-regulation of Ca^2+ ^mediated pro-inflammatory responses of PAF-activated neutrophils.

**Conclusion:**

Although generally considered to initiate and/or amplify intracellular signalling cascades which activate and sustain the pro-inflammatory activities of neutrophils and other cell types, the findings of the current study have identified a potentially important physiological, anti-inflammatory function for PKC, at least in neutrophils.

## Background

Chemoattractants, including the bioactive phospholipid, platelet-activating factor (PAF), interact with G-protein coupled receptors on the plasma membrane of human neutrophils to activate phospholipase C (PLC), which is followed by rapid and transient increases in cytosolic calcium concentrations [1,2]. Mobilization of the cation from intracellular stores is dependent on the PLC-mediated hydrolysis of membrane phospholipids, which generates inositol triphosphate (IP_3_) and diacylglycerol (DAG). IP_3 _interacts with its receptors on the membranes of calcium storage vesicles releasing Ca^2+ ^into the cytosol [3]. The intracellular concentration of IP_3 _peaks at about 10 - 15 sec following receptor ligation [2] and then declines towards basal levels consequent to both down-regulation of PLC activity [4] and intracellular metabolism of IP_3 _by phosphomonoesterases [5-8].

Although PLC activity is modulated by depletion of enzyme substrate [4], and decay of receptor-mediated signaling [4], it has also been proposed that in some cell types, namely vascular endothelial cells [9] and platelets [10], protein kinase C (PKC) negatively regulates PLC. Diacylglycerol (DAG) and Ca^2+^, both downstream products of PLC, activate PKC, which in turn, completes a negative feedback loop by inhibiting PLC. The existence and physiologic consequences of cross-talk between PKC and PLC in activated human neutrophils has, however, received little attention despite the potential of this mechanism to expedite restoration of Ca^2+ ^homeostasis and attenuate the Ca^2+^-dependent pro-inflammatory activities of these cells.

In the current study, we have utilized two selective PKC inhibitors to probe the interactions of PKC with PLC by determining the effects of these agents on intracellular IP_3 _concentrations, cytosolic calcium fluxes and Ca^2+^-dependent production of leukotriene B_4 _by PAF-activated neutrophils. Our results are compatible with a mechanism whereby PKC negatively modulates the activity of PLC, attenuating IP_3 _production and promoting the clearance of cytosolic Ca^2+^, with associated decreased production of LTB_4_.

## Materials and methods

### Chemicals and reagents

The highly selective protein kinase C inhibitor, GF10903X, was purchased from Tocris Cookson Ltd, UK. Unless indicated all other chemicals and reagents were obtained from the Sigma Chemical Co., St Louis, MO, USA. Both agents were dissolved in dimethyl sulphoxide (DMSO) to give stock concentrations of 0.8 mM and 1 mM for staurosporine and GF10903X respectively. The maximum DMSO concentration in each assay system was 0.2% and appropriate solvent controls were included for each series of experiments.

### Neutrophils

Purified human neutrophils were prepared from heparinised venous blood (five units of preservative-free heparin per ml of blood) from healthy adult volunteers. Neutrophils were separated from mononuclear leukocytes by centrifugation on Histopaque-1077 (Sigma Diagnostics) cushions at 400 × *g *for 25 min at room temperature. The resultant neutrophil fraction was removed by sequential sedimentation with 3% gelatin in order to remove most of the erythrocytes. Following centrifugation (280 × *g *at 10°C for 10 min), residual erythrocytes were removed by selective lysis with 0.84% ammonium chloride at 4°C for 10 min. The neutrophils, which were routinely of high purity (>90%) and viability (>95%), were resuspended to 1 × 10^7^.ml^-1 ^in phosphate-buffered saline (PBS 0.15 M, pH 7.0) and held on ice until used.

### Spectrofluorimetric measurement of cytosolic Ca^2+^

Fura-2/AM was used as the fluorescent, Ca^2+^-sensitive indicator for these experiments. Neutrophils (1 × 10^7^.ml^-1^) were incubated with fura-2/AM (2 μM) for 30 min at 37°C in PBS, washed and resuspended in indicator-free Hank's balanced salt solution (HBSS, pH 7.4), containing 1.25 mM CaCl_2_. The fura-2-loaded cells (2 × 10^6^.ml^-1^) were then preincubated for 10 min at 37°C in the absence or presence of the PKC inhibitors (staurosporine at 400 nM, or GF10903X at 0.5 and 1 μM), after which they were transferred to disposable reaction cuvettes, which were maintained at 37°C in a Hitachi 650 10S fluorescence spectrophotometer with excitation and emission wavelengths set at 340 and 500 nm respectively. After a stable baseline was obtained (± 1 min), the neutrophils were activated by addition of platelet-activating factor (PAF) at final concentrations of 20 and 200 nM.

A second chemoattractant, N-formyl-L-methionyl-L-leucyl-L-phenylalanine (FMLP, 1 μM, final) was used in a limited series of confirmatory experiments during which neutrophils were activated in the presence or absence of GF10903X (1 μM).

To determine the effects of the PKC inhibitors on cytosolic Ca^2+ ^concentrations, uncomplicated by Ca^2+ ^influx from extracellular reservoirs, the cells were treated with the Ca^2+^-chelating agent, ethylene glycol-bis (β-aminoethyl ether)N,N,N' N'-tetraacetic acid (EGTA, 10 mM), added to the cells 1 min prior to PAF (200 nM).

Additional experiments were performed with U73122 (2 μM), a selective inhibitor of phospholipase C, added to the cells 10 - 15 sec after PAF (200 nM), when peak cytosolic Ca^2+ ^concentrations had been reached, in the presence or absence of the PKC inhibitors staurosporine (400 nM) and GF10903X (1 μM). This experimental design was used to determine whether the putative target of PKC (following maximal mobilization of stored Ca^2+^) is PLC or the intracellular phosphomonoesterases which metabolize IP_3_.

Further experiments were conducted to investigate the effects of the test agents on the rates of resequestration of cytosolic Ca^2+ ^into storage vesicles mediated by the cAMP-sensitive endomembrane Ca^2+^-ATPase. Fura-2-loaded cells were preincubated at 37°C with staurosporine (400 nM) or GF10903X (0.5 and 1 μM) for 5 min followed by addition of the phosphodiesterase 4 inhibitor, rolipram (2 μM), for 3 min prior to activation of the cells with PAF (20 nM), and the subsequent alterations in fura-2 fluorescence monitored over a 5 min time period.

### Mn^2+ ^quenching of fura-2 fluorescence

Cells loaded with fura-2 as described above were activated with PAF (20 and 200 nM) in HBSS containing 300 μM MnCl_2 _(added 5 min prior to PAF) and fluorescence quenching as a measure of Ca^2+ ^influx was monitored at an excitation wavelength of 360 nm, which is an isosbestic wavelength, and at an emission wavelength of 500 nm [11]. This procedure was used to investigate the effects of GF10903X (0.5 and 1 μM) added to the cell suspensions 8 min before activation, on the rate and magnitude of Ca^2+ ^influx.

### Radiometric assessment of Ca^2+ ^fluxes

^45^Ca^2+ ^(Calcium-45 chloride, specific activity 18.53 mCi.mg^-1^, Perkin Elmer Life Sciences, Inc.) was used as tracer to label the intracellular Ca^2+ ^pool and to monitor Ca^2+ ^fluxes in resting and PAF-stimulated neutrophils. In the assays of Ca^2+ ^influx and efflux described below, the radiolabeled cation was used at a fixed, final concentration of 2 μCi.ml^-1^, and the final assay volumes were 5 ml containing a total of 1 × 10^7 ^neutrophils. The standardization of the procedures used to load the cells with ^45^Ca^2+^, as well as a comparison with oil-based methods for the separation of labeled neutrophils from unbound isotope, have been described previously [12].

### Efflux of ^45^Ca^2+ ^from neutrophils

Neutrophils (1 × 10^7^.ml^-1^) were loaded with ^45^Ca^2+ ^(2 μCi.ml^-1^) for 30 min at 37°C in HBSS which was free of unlabeled Ca^2+^. The cells were then pelleted by centrifugation, washed once with, and resuspended in ice-cold Ca^2+^-replete HBSS and held on ice until use, which was always within 10 min of completion of loading with ^45^Ca^2+^. The ^45^Ca^2+^-loaded neutrophils (2 × 10^6^.ml^-1^) were then preincubated for 10 min at 37°C in Ca^2+^-replete HBSS, in the presence and absence of GF10903X (1 μM), followed by addition of PAF (20 nM) and measurement of the efflux of ^45^Ca^2+ ^over 5 min. The reactions were terminated by the addition of 10 ml ice-cold, Ca^2+^-replete HBSS to the tubes which were then transferred to an ice-bath. The cells were then pelleted by centrifugation at 400 × *g *for 5 min followed by washing with 15 ml ice-cold, Ca^2+^-replete HBSS and the cell pellets finally dissolved in 0.5 ml of 0.5% triton X-100/0.1 M NaOH and the radioactivity assessed in a liquid scintillation spectrometer. Control, cell-free systems (HBSS and ^45^Ca^2+ ^only) were included for each experiment and these values were subtracted from the relevant neutrophil-containing systems. These results are presented as the percentage of cell-associated radiolabeled cation extruded from the cells.

### Influx of ^45^Ca^2+ ^into PAF-activated neutrophils

To measure the net influx of ^45^Ca^2+ ^into PAF-activated neutrophils, uncomplicated by concomitant efflux of the radiolabeled cation, the cells were loaded with cold, Ca^2+^-replete HBSS for 30 min at 37°C, after which the cells were pelleted by centrifugation, then washed once with, and resuspended in ice-cold Ca^2+^-free HBSS and held on ice until used. Pre-loading with cold Ca^2+ ^was undertaken to minimize spontaneous uptake of ^45^Ca^2+ ^(unrelated to PAF activation) in the influx assay. The Ca^2+^-loaded neutrophils (2 × 10^6^.ml^-1^), were then incubated for 10 min in the presence or absence of GF10903X (1 μM) at 37°C in HBSS containing 25 μM cold carrier Ca^2+ ^(as CaCl_2_), followed by simultaneous addition of PAF (20 or 200 nM) and ^45^Ca^2+ ^(2 μCi/ml) or ^45^Ca^2+ ^only to control, unstimulated systems. Influx of ^45^Ca^2+ ^into PAF-activated neutrophils was then monitored over a 5 min period, after which influx is complete and compared with the uptake of the radiolabeled cation by identically processed, unstimulated cells as described above.

### Inositol triphosphate (IP_3_)

Neutrophils at a concentration of 5 × 10^6^.ml^-1 ^in Ca^2+^-replete HBSS were preincubated for 10 min at 37°C in the presence or absence of GF10903X (1 μM), followed by the addition of PAF (20 or 200 nM) or FMLP (1 μM) in a final volume of 2 ml, after which the reactions were terminated and the IP_3 _extracted by the addition of 0.4 ml of 20% perchloric acid at 10 and 20 sec after addition of the chemoattractant, and the tubes transferred to an ice bath. These incubation times coincide with the early peak IP_3 _responses (10 sec) of PAF-activated neutrophils, as well as the subsequent decline (20 sec) towards basal levels which are reached at around 60 sec [1,2], determined in a series of preliminary experiments. In an additional series of experiments, the effects of the PKC activator, phorbol 12-myristate 13-acetate (PMA, 50 ng/ml final, added 2 min before PAF) on the IP_3 _responses of PAF (200 nM)-activated cells in the absence and presence of GF10903X (1 μM) were investigated.

Following 20 min incubation on ice, the tubes were centrifuged at 2000 × *g *for 15 min and the supernatants removed and brought to pH 7.5 with 5N KOH, followed by centrifugation at 2000 × *g *for 15 min to remove precipitated perchloric acid. The supernatants were assayed using the inositol-1,4,5-triphosphate [^3^H] radioreceptor assay procedure (Perkin Elmer Life Sciences, Inc., Boston, MA, USA), which is a competitive ligand binding assay, and the results expressed as pmol IP_3_/10^7 ^cells.

### Measurement of LTB_4_

A competitive binding enzyme immunoassay procedure (Correlate-EIA™; Assay Designs Inc., Ann Arbor, MI, USA) was used to measure LTB_4 _in the supernatants of neutrophils activated with PAF (20 and 200 nM) in the absence or presence of GF10903X (1 μM). Neutrophils (2 × 10^6^.ml^-1^, final) in HBSS were preincubated for 10 min at 37°C with the test agent after which PAF was added to the cells and the reactions stopped after 3 min incubation at 37°C (predetermined in preliminary time-course experiments) by the addition of an equal volume of ice-cold HBSS to the tubes which were then held in an ice-bath prior to pelleting the cells by centrifugation. The cell-free supernatants were then assayed for LTB_4 _using the enzyme immunoassay (EIA) procedure. Supernatants from cells activated with PAF were diluted 1:4 prior to assay. These results are expressed as picograms (pg)/10^7 ^cells.

### Statistical Analysis

The results of each series of experiments (*n *values represent the number of separate experiments in each series for which cells from a minimum of 3 different donors were used) are expressed as the mean value ± standard error of the mean (S.E.M.), with the exception of the fura-2/AM experiments for which the traces are also presented. Levels of statistical significance were calculated using paired Student's *t *test when comparing two groups, or by analysis of variance (ANOVA) with subsequent Tukey-Kramer multiple comparisons test for multiple groups. A *P*-value < 0.05 was considered significant.

## Results

### Effects of staurosporine and GF10903X on the fura-2 responses of PAF- or FMLP-activated neutrophils

**Figure 1:**
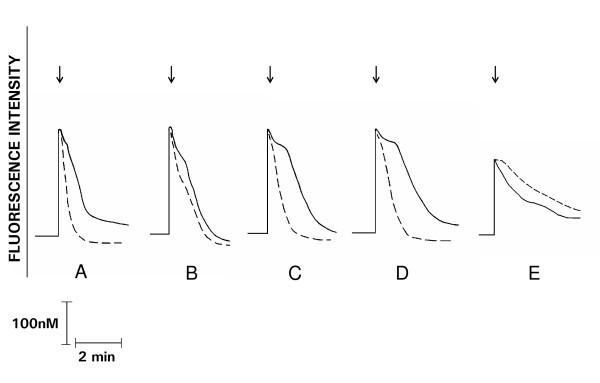
**Fura-2 fluorescence responses of PAF (20 nM)-activated neutrophils (A), pretreated with staurosporine 400 nM (B), GF10903X 0.5 μM (C) and 1 μM (D), in the presence (_ _ _) or absence (____) of rolipram (2 μM), as well as those of FMLP (1 μM)-activated cells (E), with (_ _ _) and without (____) GF10903X (1 μM)**. These are traces from a single representative experiment with a total of 3 8 in each series. Addition of the chemoattractant is denoted by the arrow (↓).

**Figure 2:**
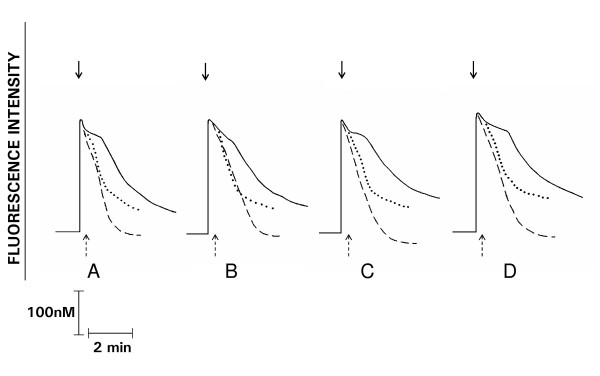
**Fura-2 fluorescence responses of PAF (200 nM)-activated neutrophils (A), pre-treated with staurosporine 400 nM (B), GF10903X 0.5 μM (C), and 1 μM (D) in the presence (_ _ _), or absence (____) of EGTA or U73122 (2 μM) (.....) added 10 - 15 sec after PAF**. These are traces from a single representative experiment with a total of 4 - 12 in each series. The arrows denote addition of PAF (↓) or U73122 (↑).

These results are shown in Figures 1 and 2. Exposure of neutrophils to PAF (20 nM) was accompanied by an abrupt increase in fura-2 fluorescence intensity, typical of G-protein-coupled receptor activation of phospholipase C and inositol triphosphate-mediated release of Ca^2+ ^from intracellular stores. Peak fluorescence intensity declined within a few seconds and continued to decrease steadily towards resting levels. Pretreatment of the cells with the PKC inhibitors, staurosporine and GF10903X, did not alter the magnitude of the peak fluorescence, but was associated with a sustained elevation in peak cytosolic Ca^2+ ^concentrations that declined towards resting levels at significantly slower rates than those observed for control systems (Figure 1).

Activation of neutrophils with FMLP resulted in an abrupt increase in fura-2 fluorescence intensity which coincided with the rise in cytosolic Ca^2+ ^concentrations, and quickly subsided, returning to base-line after several minutes. In the presence of GF10903X, the peak fluorescence intensity was not altered, but was followed by a sustained plateau phase of about 30 sec which subsequently declined towards basal levels at a significantly slower rate than that observed with control systems (Figure 1).

Addition of PAF at the higher concentration (200 nM) to neutrophils was accompanied by an abrupt increase in fura-2 fluorescence intensity due to elevation in the cytosolic Ca^2+ ^concentration which also peaked rapidly, but which was followed by a sustained plateau phase lasting about 1 min with a subsequent gradual decline in fluorescence intensity towards basal levels (Figure 2). In the presence of staurosporine or GF10903X, the magnitudes of peak fluorescence intensity were not altered, but the duration of the plateau phase was significantly prolonged and the subsequent gradual decline in fluorescence intensity was slower than that observed for control systems.

### Effects of EGTA on fura-2 responses

In the presence of the Ca^2+^-chelating agent, EGTA, addition of PAF (200 nM), was also accompanied by the characteristic abrupt increase in fura-2 fluorescence, which subsequently declined rapidly towards basal levels without the sustained elevation in fluorescence intensity observed in the absence of EGTA (Figure 2). Treatment of neutrophils with the PKC inhibitors did not alter the magnitude of the initial peak cytosolic Ca^2+ ^concentrations, but the rate of decline towards basal levels was slower. The effects of these agents on the rate of decline in fluorescence intensity were less pronounced than those observed in the absence of EGTA (preserved extracellular Ca^2+ ^reservoirs). GF10903X (1 μM) had no effect on thapsigargin-mediated Ca^2+ ^release from intracellular storage vesicles (results not shown).

### Effects of U73122 on fura-2 responses

The effects of the phospholipase C (PLC) inhibitor, U73122 (2 μM) added to neutrophils 10 - 15 sec following addition of PAF (200 nM), are shown in Figure 2. At this concentration, U73122 abolishes receptor-mediated Ca^2+ ^mobilization and IP_3 _generation by neutrophils [13], which were confirmed in a series of preliminary experiments (not shown). Addition of U73122 resulted in a rapid decline in fluorescence intensity with marked attenuation of the prolonged plateau phase. Similarly, in the presence of the PKC-inhibitors, addition of U73122 resulted in an almost immediate decline in fura-2 fluorescence intensity.

### Effects of rolipram on fura-2 responses

These results are shown in Figure 2. Neutrophils were treated with the phosphodiesterase inhibitor, rolipram in order to investigate the effects of the PKC inhibitors on the rates of resequestration of Ca^2+ ^into storage vesicles mediated by the protein kinase A (PKA)-sensitive Ca^2+^-endomembrane ATPase. In the presence of rolipram, cAMP accumulates in neutrophils, activating PKA with consequent upregulation of the activity of the endomembrane Ca^2+^-ATPase [14]. Neutrophils were pretreated with the PKC inhibitors for 5 min, followed by rolipram for 3 min. The magnitude of the peak fluorescence response was not altered by rolipram, but the rate of decline in cytosolic Ca^2+ ^concentrations were markedly accelerated following attainment of peak fluorescence. Similar effects of rolipram were observed in neutrophils pretreated with the PKC inhibitors, suggesting that these agents do not interfere with endomembrane ATPase-mediated resequestration of Ca^2+ ^into storage vesicles.

The consolidated data for all of the fura-2 fluorescence experiments described above are shown in Tables 1 and 2.

**Table 1:** Effects of staurosporine and GF10903X, in the presence or absence of rolipram, on cytosolic Ca^2+ ^concentrations of PAF- activated neutrophils, as well as the effects of GF10903X on cytosolic Ca^2+ ^concentrations in FMLP-stimulated cells.

**System**	**Peak** **(nM)**	**Plateau** **(min)**	**Magnitude of decrement (nM) from peak measured at:**	
			**1 min**	**2 min**
			
PAF (20 nM) Control (n = 6)	278 ± 16	0.4 ± 0.05	108 ± 10	168 ± 12
Staurosporine (400 nM) (n = 4)	290 ± 8	1.0 ± 0.05*	52 ± 4*	160 ± 15
GF10903X (0.5 μM) (n = 7)	258 ± 4	1.0 ± 0.04*	32 ± 2*	124 ± 5*
GF10903X (1 μM) (n = 7)	266 ± 10	1.1 ± 0.02*	24 ± 3*	104 ± 4*
Rolipram (2 μM) (n = 4)	274 ± 4	0.13 ± 0.03	188 ± 4	216 ± 4
Staurosporine + Rolipram (n = 4)	286 ± 6	0.2 ± 0.06	144 ± 8**	200 ± 8
GF10903X (0.5 μM) + Rolipram (n = 4)	286 ± 7	0.17 ± 0.03	164 ± 14**	196 ± 10**
GF10903X (1 μM) + Rolipram (n = 4)	282 ± 8	0.27 ± 0.03	152 ± 18**	200 ± 11**
FMLP (1 μM) Control (n = 6)	285 ± 7	0.1 ± 0.01	94 ± 3	142 ± 9
GF10903X (1 μM) (n = 11)	278 ± 4	0.53 ± 0.05*	54 ± 3*	107 ± 5*

The results are expressed as the mean percentage of control ± S.E.M. * *P *< 0.05 for comparison with the untreated control system and **P < 0.05 for comparison with rolipram-treated cells. Basal Ca^2+ ^concentrations were 80 ± 8 nM.

**Table 2:** Effects of staurosporine and GF10903X, in the presence or absence of EGTA, on cytosolic Ca^2+ ^concentrations of PAF-activated neutrophils, as well as the effects of U73122 added 10 - 15 sec after PAF on cytosolic Ca^2+ ^concentrations.

**System**	**Peak** **(nM)**	**Plateau** **(min)**	**Magnitude of decrement (nM) from peak measured at:**			
			**1 min**	**2 min**	**3 min**	**5 min**
			
PAF (200 nM) Control (n = 12)	270 ± 8	0.8 ± 0.06		80 ± 5	116 ± 6	168 ± 8
Staurosporine (400 nM) (n = 6)	290 ± 16	1.03 ± 0.08		60 ± 11*	92 ± 18	136 ± 20
GF10903X (0.5 μM) (n = 9)	274 ± 9	1.11 ± 0.05*		52 ± 5*	72 ± 6*	112 ± 7*
GF10903X (1 μM) (n = 9)	270 ± 8	1.2 ± 0.07*		44 ± 6*	64 ± 8*	100 ± 8*
EGTA (n = 10)	246 ± 4		100 ± 5	164 ± 6		
EGTA + Staurosporine (n = 7)	246 ± 8		76 ± 5^+^	136 ± 9^+^		
EGTA + GF10903X (0.5 μM) (n = 7)	238 ± 10		78 ± 5^+^	132 ± 6^+^		
EGTA + GF10903X (1 μM) (n = 7)	246 ± 9		76 ± 3^+^	132 ± 6^+^		
U73122 (2 μM) (n = 4)	266 ± 11	0.4 ± 0.05	120 ± 9	140 ± 7		
U73122 + Staurosporine (n = 3)	290 ± 12	0.43 ± 0.06	112 ± 15	132 ± 9		
U73122 + GF10903X (0.5 μM) (n = 3)	278 ± 11	0.43 ± 0.07	92 ± 16	116 ± 9		
U73122 + GF10903X (1 μM) (n = 3)	262 ± 8	0.52 ± 0.05**	92 ± 11	108 ± 8**		

The results are expressed as the mean percentage of control ± S.E.M. * *P *< 0.05 for comparison with the untreated control system and ^+^*P *< 0.05 or ** *P *< 0.05 for comparison with EGTA or U73122-treated neutrophils, respectively. Basal Ca^2+ ^concentrations were 80 ± 8 nM.

### Mn^2+ ^quenching of fura-2 fluorescence

**Figure 3:**
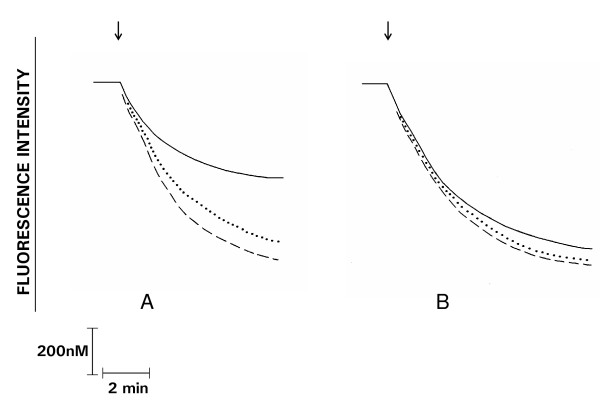
**Effects of GF10903X 0.5 μM (.....) or 1 μM (_ _ _) on the Mn^2+ ^quenching of fura-2 fluorescence assay in PAF 20 nM (A)- or 200 nM (B)-activated neutrophils**. PAF was added as indicated (↓). These are traces from a single representative experiment with a total of 5 - 8 in each series.

These results are shown in Figure 3 and Table 3. In control cells, the decrease in fluorescence intensity, which indicates influx of Ca^2+^, occurred almost immediately after addition of PAF (20 and 200 nM). An initial abrupt linear decrease in fluorescence intensity over 2 - 3 min, of greater magnitude at the higher concentration of PAF, was followed by a slower decline for a further 2 - 3 min. In the presence of the PKC inhibitors, addition of PAF (20 nM) was followed by a rapid decline in fura-2 fluorescence intensity of significantly greater magnitude (measured 1, 3 and 5 min after addition of the chemoattractant) than that observed with untreated cells. In the presence of the PKC inhibitors, addition of PAF (200 nM), resulted in a slight, but insignificant increase in the magnitude of decline in fura-2 fluorescence.

**Table 3:** Effects of GF10903X on the Mn^2+ ^quenching of fura-2 fluorescence in PAF-activated neutrophils, as well as the effects of GF10903X on ^45^Ca^2+ ^uptake by PAF-stimulated cells.

**System**	**Magnitude of the decrement in fura-2 fluorescence intensity in the presence of Mn^2+ ^(cm):**			**^45^Ca^2+ ^Uptake(pmol ^45^Ca^2+^/10^7 ^cells)**	
	**1 min**	**3 min**	**5 min**	**3 min**	**5 min**
	
PAF (20 nM) Control	1.8 ± 0.13	3.4 ± 0.2	4.2 ± 0.3	46 ± 6	53 ± 4
GF10903X (0.5 μM)	2.2 ± 0.13*	5.3 ± 0.3*	6.5 ± 0.2*		
GF10903X (1 μM)	2.1 ± 0.1*	6.0 ± 0.23*	7.3 ± 0.3*	125 ± 18*	116 ± 16*
PAF (200 nM) Control	2.6 ± 0.16	6.3 ± 0.6	7.4 ± 0.7	48 ± 12	112 ± 5
GF10903X (0.5 μM)	2.9 ± 0.35	6.9 ± 0.6	8.3 ± 0.6		
GF10903X (1 μM)	2.5 ± 0.26	6.5 ± 0.7	8.2 ± 0.7	41 ± 2	105 ± 4

The results of 5 - 8 experiments are expressed as the mean percentage magnitude of decrement (Mn^2+ ^quenching) or mean percentage of control (^45^Ca^2+ ^uptake) ± S.E.M. * *P *< 0.05 for comparison with the untreated control system.

The rate and magnitude of decline in fura-2 fluorescence for neutrophils activated with FMLP (1 μM), was significantly increased in the presence of GF10903X (1 μM), (results not shown).

### Effects of the PKC inhibitors on the net influx and net efflux of Ca^2+^

The magnitudes of net influx of Ca^2+ ^following activation of neutrophils with 20 and 200 nM PAF are shown in Table 3. Treatment of neutrophils with GF10903X significantly increased the magnitude of store-operated influx of Ca^2+ ^following activation of the cells with PAF at a concentration of 20 nM. No significant differences were observed for neutrophils activated with higher concentrations of PAF (200 nM). These results correspond closely with those obtained by means of the Mn^2+ ^quenching of fura-2 fluorescence assays.

The net efflux of Ca^2+ ^from PAF (20 nM)-activated neutrophils measured 5 min following addition of the chemoattractant was 4 ± 2% of the total amount of cell-associated radiolabelled Ca^2+ ^and this increased significantly to 12 ± 2% for cells pretreated with GF10903X, (*P *< 0.05 for comparison with the untreated control system).

### Effects of the PKC inhibitors on inositol triphosphate production

These results are shown in Table 4. IP_3 _concentrations increased significantly following exposure of neutrophils to PAF (20 and 200 nM) or FMLP (1 μM), peaking at 10 sec after addition of the chemoattractant. Pre-incubation of the cells with GF10903X (1 μM) resulted in significant increases in IP_3 _concentrations.

**Table 4:** Effects of GF10903X (1 μM) on the IP_3 _concentrations of chemoattractant-activated neutrophils.

**System**	**IP_3 _concentrations (pg/ml) measured at:**	
	**10 sec**	**20 sec**
	
PAF (20 nM)	46 ± 3.5	N.D.
GF10903X + PAF (20 nM)	75 ± 5.5*	N.D.
PAF (200 nM)	82 ± 8.3	65 ± 17
GF10903X + PAF (200 nM)	136 ± 6.4*	120 ± 2.3*
FMLP (1 μM)	58 ± 16	N.D.
GF10903X + FMLP	96 ± 8*	N.D.

The results of 4 experiments are expressed as the mean IP_3 _concentration ± S.E.M., rising from basal values of 35 ± 2 pg/ml, for PAF (20 and 200 nM) or FMLP (1 μM)- treated cells, in the absence or presence of GF10903X. * *P *< 0.05 for comparison with the untreated system.

### Effects of GF10903X on LTB_4 _production by activated neutrophils

LTB_4 _production by PAF (20 nM)-activated neutrophils was markedly increased in the presence of GF10903X from 175 ± 31 to 794 ± 51 pg/10^7 ^cells in the absence or presence of the PKC inhibitor respectively (*P *< 0.01), rising from a basal value of 24 ± 6 pg/10^7 ^for resting cells.

## Discussion

The results of the current study have identified a role for PKC in promoting restoration of Ca^2+ ^homeostasis and down-regulation of Ca^2+^-dependent pro-inflammatory activity to chemoattractant-activated human neutrophils. Notwithstanding those which target IP_3 _and its receptor, well-characterized mechanisms which promote efficient clearance of Ca^2+ ^from the cytosol of activated neutrophils include: i) the electrical gradient created by the membrane depolarizing action of NADPH oxidase that restricts the influx of Ca^2+ ^via store-operated Ca^2+ ^channels [15-17] and ii) the combined action of two ATP-driven Ca^2+ ^pumps, namely the Ca^2+^-resequestering endomembrane Ca^2+^- ATPase and the plasma membrane Ca^2+^-ATPase, that actively transports Ca^2+ ^out of the cell [18,19]. However, based on the following observations, neither NADPH oxidase nor either of the Ca^2+ ^pumps were considered to be putative targets for PKC in our experimental setting. Firstly, PAF, at the concentrations used in this study, does not activate NADPH oxidase [20], effectively excluding alterations in membrane potential as a mechanism for the prolonged cytosolic Ca^2+ ^transients observed with the PKC inhibitors. Secondly, the apparent enhanced Ca^2+ ^efflux in the presence of GF10903X is not compatible with inhibition of the plasma membrane-associated Ca^2+^-ATPase, which is upregulated by sustained elevations in cytosolic Ca^2+ ^concentrations [21]. Thirdly, the sensitivity of the endomembrane Ca^2+^-ATPase to rolipram was preserved in PAF-activated neutrophils pretreated with the PKC-inhibitors, suggesting that these agents do not significantly interfere with the refilling of Ca^2+ ^stores.

From a mechanistic perspective however, treatment of neutrophils with GF10903X significantly elevated and prolonged the concentrations of the intracellular second messenger, IP_3_, in chemoattractant-activated neutrophils. The apparent doubling of IP_3 _concentrations in the presence of the PKC inhibitor observed in the current study likely maintains IP_3 _receptors in an open state for longer periods, facilitating sustained Ca^2+ ^release by promoting shuttling of the cation between the stores and the cytosol [22].

Experiments performed in the presence of the extracellular Ca^2+^-chelating agent, EGTA, support this contention, as delayed Ca^2+ ^clearance in the presence of the PKC inhibitors persisted in this setting, and could not be attributed to enhanced Ca^2+ ^influx. Previous reports have suggested that PKC may modulate PAF-mediated activation of PLC by promoting desensitization of the PAF receptor [23]. This is an unlikely mechanism in human neutrophils, as similar effects of the PKC inhibitors were observed when the cells were activated with the formyl peptide, FMLP, a ligand which interacts with receptors considered resistant to PKC-mediated phosphorylation [24,25].

Sustained activation of IP_3 _receptors at higher concentrations of IP_3 _not only mobilizes stored Ca^2+^, but also activates store-operated influx mechanisms [26]. In addition, IP_3 _activates Ca^2+ ^channels independently of the filling state of Ca^2+ ^stores [27]. These IP_3-_dependent mechanisms are also likely to contribute to the prolonged cytosolic Ca^2+ ^transients in GF10903X/staurosporine-treated cells. In support of this contention, the magnitudes of Ca^2+ ^reuptake determined by means of both the Mn^2+ ^quenching of fura-2 fluorescence assay and radiometric procedure were markedly increased in the presence of the PKC inhibitors when neutrophils were activated with PAF at 20 nM, but less so at higher concentrations (200 nM). Ca^2+ ^influx mechanisms are clearly submaximally activated at lower PAF concentrations and can be increased by potentiation of the IP_3 _signal.

The magnitude and duration of the IP_3 _response to chemoattractants reflect a balance between PLC activity and IP_3 _metabolism by intracellular phosphomonoesterases [5-8]. Because PKC has been reported to activate 5'-phosphomonoesterases that metabolize IP_3 _[8], we also investigated the effects of addition of U73122, a PLC inhibitor, to the cells 10 - 15 sec after PAF, when Ca^2+ ^mobilization and IP_3 _generation are complete. U73122 markedly attenuated the prolongation of cytosolic Ca^2+ ^transients in the presence of the PKC inhibitors, suggesting that persistent PLC activity is primarily responsible for the exaggerated IP_3 _production. Nevertheless, impaired activation of 5'-phosphomonoesterases cannot be conclusively excluded.

Further evidence, albeit indirect, that PKC down-regulates PLC activity, is suggested by our previous observations that co-activation of neutrophils with PAF and a phorbol ester, a direct activator of PKC, attenuates PAF-mediated prolongation of peak cytosolic Ca^2+ ^transients [20].

To determine the functional consequences of inactivation of PKC on the Ca^2+^-dependent pro-inflammatory activities of neutrophils, we measured the effect of GF10903X on PAF-activated leukotriene B_4 _(LTB_4_) production. Production of this highly pro-inflammatory eicosanoid was markedly enhanced by treatment of the cells with the PKC inhibitor, underscoring the role of PKC in down-regulating the Ca^2+^-dependent pro-inflammatory activities of neutrophils. LTB_4 _recruits and activates not only neutrophils and other types of inflammatory cells, but also amplifies IP_3 _production via a positive feedback autocrine loop, whereby LTB_4 _released from the cell, interacts with its receptor on the plasma membrane to activate PLC [28,29]. Consequently, IP_3 _generation is sustained and this in turn may exaggerate the pro-inflammatory activity of neutrophils.

## Conclusion

In conclusion, the current study has demonstrated that PKC down-regulates Ca^2+^-dependent pro-inflammatory responses of chemoattractant-activated neutrophils, presumably by phosphorylative inactivation of PLC, resulting in termination of IP_3 _production. This in turn, favours rapid restoration of Ca^2+ ^homeostasis and attenuation of pro-inflammatory activity, a potentially important physiological mechanism of endogenous control of neutrophil inflammation.

## Competing interests

The authors declare that they have no competing interests.

## Authors' contributions

GRT designed and conducted cytosolic calcium experiments and wrote the manuscript; AJT prepared neutrophil suspensions and assisted with experiments; HCS and RC conducted leukotriene B_4 _and IP_3 _experiments; LP assisted in formatting and editing the manuscript and RA assisted with experiments, interpretation of results and editing the manuscript.

All of the authors have read and approved the manuscript.
